# LightEyes: A Lightweight Fundus Segmentation Network for Mobile Edge Computing

**DOI:** 10.3390/s22093112

**Published:** 2022-04-19

**Authors:** Song Guo

**Affiliations:** School of Information and Control Engineering, Xi’an University of Architecture and Technology, Xi’an 710055, China; guosong@xauat.edu.cn

**Keywords:** lightweight network, fast semantic segmentation, mobile edge computing, fundus image

## Abstract

Fundus is the only structure that can be observed without trauma to the human body. By analyzing color fundus images, the diagnosis basis for various diseases can be obtained. Recently, fundus image segmentation has witnessed vast progress with the development of deep learning. However, the improvement of segmentation accuracy comes with the complexity of deep models. As a result, these models show low inference speeds and high memory usages when deploying to mobile edges. To promote the deployment of deep fundus segmentation models to mobile devices, we aim to design a lightweight fundus segmentation network. Our observation comes from the fact that high-resolution representations could boost the segmentation of tiny fundus structures, and the classification of small fundus structures depends more on local features. To this end, we propose a lightweight segmentation model called LightEyes. We first design a high-resolution backbone network to learn high-resolution representations, so that the spatial relationship between feature maps can be always retained. Meanwhile, considering high-resolution features means high memory usage; for each layer, we use at most 16 convolutional filters to reduce memory usage and decrease training difficulty. LightEyes has been verified on three kinds of fundus segmentation tasks, including the hard exudate, the microaneurysm, and the vessel, on five publicly available datasets. Experimental results show that LightEyes achieves highly competitive segmentation accuracy and segmentation speed compared with state-of-the-art fundus segmentation models, while running at 1.6 images/s Cambricon-1A speed and 51.3 images/s GPU speed with only 36k parameters.

## 1. Introduction

Fundus is the only human body structure that can be observed without causing trauma to the human body [1]. The conditions of fundus can reflect relevant pathological features from different angles and aspects, and have important value for the study of cardiovascular and cerebrovascular diseases [2,3]. At present, fundus examination is mainly based on the manual analysis of fundus images by ophthalmologists.

Retinal vasculopathy and other pathologies such as microaneurysm (MA), hard exudate (EX), and soft exudate can be directly observed through the fundus image. Among them, MA and EX play an important role in the grading of diabetic retinopathy (DR), which is the leading cause for blindness in adults and is a serious public problem all over the world. Specially, the appearance of MA is the main marker of mild non-proliferative DR. If the MA can be detected by fundus image examination, effective interventions can be performed to reduce the risk of DR. Moreover, EX is not only related to the diagnosis of DR, but also to the diagnosis of diabetic macular edema and age-related macular degeneration, both of which are serious diseases of the eyes. In addition, the morphological changes of retinal vessels, such as diameter and curvature, can be used as the basis for early screening of hypertension, stroke, coronary heart disease, and other diseases [4]. Therefore, it is necessary to analyze fundus image at the early clinical stage. However, ophthalmologists are seriously inadequate, compared with hundreds of millions of patients awaiting treatment, posing difficulties for large-scale fundus examinations. The problem is much more serious in the regions with poor medical conditions. In conclusion, a fast and automated fundus image segmentation method is required.

However, most existing fundus segmentation models use VGGNet [5] or ResNet [6] as the backbone, which may incur several questions. (1) The inference speed of the segmentation models depends heavily on the speed of the backbone. (2) The segmentation models are difficult to be deployed to resource-limited devices due to high memory usage of the backbone. (3) The spatial detailed information is missing in the backbone, which increases the difficulty of locating the boundary of small lesions. These questions make these models show poor performance in mobile edge computing.

**Figure 1:**
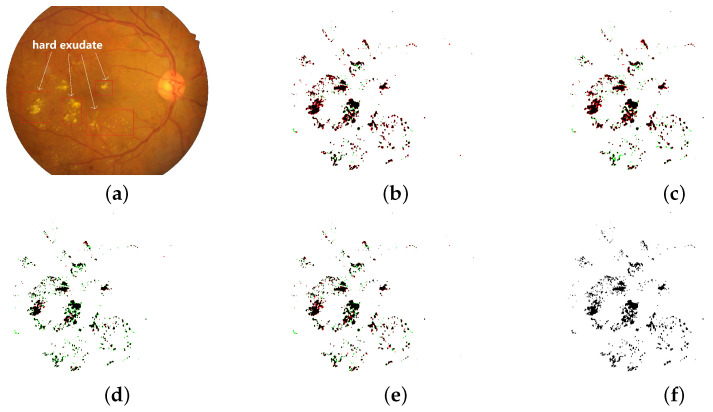
Hard exudate segmentation binary maps of the LightEyes and other methods on the IDRiD dataset (TPs are marked as black, FPs are marked as red, and FNs are marked as green. JI denotes Jaccard similarity index). (**a**) fundus image. (**b**) HED [7] (JI = 0.6663). (**c**) L-Seg [8] (JI = 0.6478) (**d**) LWENet [9] (JI = 0.6952). (**e**) LightEyes (JI = 0.7159). (**f**) ground truth.

To solve the above problems, in this paper, we propose a lightweight segmentation network, called LightEyes, for the mobile edge. LightEyes has two characteristics, which make it not only run efficiently on the mobile edge devices but also show superior segmentation performance. The first characteristic is that the encoder of the LightEyes contains no downsampling operations, so that the high resolution of features could be always preserved. This setting means that the LightEyes could segment the boundaries of the small lesions much more accurately (see Figure 1). Specifically, the encoder of the LightEyes consists of 24 convolutional layers, each with 3 × 3 kernel size, so that the receptive field of the encoder is 49 × 49, and this large of field of view ensures that the LightEyes learns semantic features rather than local features. The second characteristic of the LightEyes is that the number of convolution filters (kernels) in each convolutional layer is at most 16, far lower than the number of VGGNet and ResNet, which is about 512 or even more. The setting of a thin convolutional filter reduces the memory greatly and this makes it inference efficiently on mobile edge devices.

We evaluate the performance of the LightEyes in the microaneurysms segmentation, the exudate segmentation, and the vessel segmentation. Experimental results show that LightEyes achieves highly competitive segmentation accuracy and segmentation speed compared with state-of-the-art fundus segmentation models, while running at 1.6 images/s Cambricon-1A speed and 51.3 images/s GPU speed with only 36 k parameters.

Our contributions are summarized as follows.

1. We introduce two principles to design fundus image segmentation model and we present the LightEyes. The LightEyes aims at learning high-resolution representations without sacrificing the inference speed.
2. We conduct extensive experiments on the segmentation of three fundus structures, including the MA, the EX, and the vessels. Comparative studies reveal that, compared with other deep segmentation models and lightweight models, the LightEyes achieves better accuracy–speed trade-off.
3. We deploy the LightEyes on mobile devices. Results show that the LightEyes can process large resolution images, whereas VGGNet-based models cannot.

The remainder of this paper is organized as follows. Related works about the analysis of fundus images are introduced in Section 2. The structure of the LightEyes is described in Section 3. Experiments and analysis are given in Section 4. Discussions are given in Section 5. Conclusions are drawn in Section 6.

## 2. Related Works

In this section, recent works on the segmentation of fundus lesions and vessels are summarized (See Table 1), and then we review literature about lightweight segmentation models.

### 2.1. Fundus Lesion Segmentation

DR-related fundus lesions mainly contain hard exudate (EX), hemorrhage (HE), microaneurysm (MA), and soft exudate (SE). Existing lesion segmentation methods mainly focus on these four lesions, and could be roughly divided into two categories, namely, traditional image processing methods and deep-learning-based methods.

The main pipeline of traditional lesion recognition methods includes image preprocessing, candidate regions generation, classification, and postprocessing [10,11,12]. First, in the preprocessing stage, image enhancement algorithms are used and certain interference structures, such as bright structures and optic disc, are removed. Second, a thresholding algorithm or other method is adopted to generate candidate regions. Third, certain methods are used to classify these regions. Finally, a false positive elimination method could be used if necessary. The main disadvantage of this kind of method is the complex and non-reusable feature engineering. However, learning-based models could avoid this feature selection because the features are learned from the raw images.

Recent studies show that deep-learning-based methods achieve superior performance to traditional ones in fundus lesion segmentation [8,13]. Mo et al. proposed a patch-level ResNet-based [6] network, called FCRN, for EX segmentation [13]. FCRN follows the popular semantic segmentation framework, which consists of an encoder and a decoder [14]. In FCRN, the encoder is ResNet, and the decoder uses skip connections for multi-scale feature fusion. Guo et al. proposed a unified framework based on VGGNet for multi-lesion segmentation, called L-Seg [8]. L-Seg is an image-level model that can segment four kinds of lesions simultaneously. However, both FCRN and L-Seg use ImageNet-based classification network as the encoder, which means that they have a huge number of parameters and a high memory usage. In addition, Guo et al. proposed LWENet for EX segmentation [9], where the backbone network is newly designed, and it shows advantages in terms of the speed and the number of parameters. However, it still needs transfer learning to achieve a good performance, similar to FCRN and L-Seg. Huang et al. proposed RTN [15] for multi-lesion segmentation and it achieved an average AUC_PR of 0.7076 on the IDRiD dataset, reaching new state-of-the-art performance. Zhou et al. incorporated classification task into segmentation task and proposed a collaborative learning method [16], achieving an average AUC_PR of 0.7044 on the IDRiD dataset. Besides designing complex deep models, Sarhan utilized triplet loss for microaneurysms segmentation [17].

In conclusion, most of the existing image-level segmentation models use VGGNet or ResNet as the encoder, and the inference speed and memory usage depend heavily on the backbone. As a result, these models are not suitable to be deployed to mobile side. Meantime, there is still a lack of research in the design of the encoder for the segmentation of fundus images. Designing a new encoder meets the difficulty that no pretraining model is available, which further increases the difficulty of training using dozens of fundus images.

**Table 1:** Summary of related works (EX, SE, MA, and HE represent hard exudate, soft exudate, microaneurysms, and hemorrhage, respectively).

Authors	Year	Task	Method
Mo et al. [13]	2018	Lesion (EX)	ResNet-based deep model
Guo et al. [8]	2019	Lesion (EX, SE, MA, HE)	VGGNet-based deep model
Guo et al. [9]	2019	Lesion (EX)	Lightweight deep model
Colomer et al. [10]	2020	Lesion (EX, MA, HE)	Morphological and texture descriptors
Sarhan et al. [17]	2021	Lesion (MA)	Triplet Loss
Huang et al. [15]	2022	Lesion (EX, SE, MA, HE)	Transformer-based model
Oliveira et al. [18]	2018	Vessel	Patch-level FCN
Wu et al. [19]	2018	Vessel	Stacking multiple U-Nets
Khawaja et al. [20]	2019	Vessel	Line detector
Guo et al. [21]	2019	Vessel	Deep supervision
Jin et al. [22]	2019	Vessel	U-Net with deformable convolution
Wang et al. [23]	2020	Vessel	U-shape model with multiple decoders
Lin et al. [24]	2021	Vessel	High-resolution network

### 2.2. Fundus Vessel Segmentation

Existing vessel segmentation methods can be roughly divided into two categories: unsupervised methods and supervised methods.

Unsupervised methods do not need supervised information, and they are implemented based on human-designed rules [20,25]. However, fundus images taken from clinical environments have large intervariance, which makes unsupervised methods show poor performance since it is hard to design suitable features on such large number of images.

Supervised methods, specially deep-learning-based methods, have been fully exploited in recent years and show superior performance to unsupervised ones [21,26,27]. In this work, we only discuss segmentation-network-based methods. Segmentation-network-based methods could be further divided into image-level segmentation network and patch-level segmentation network, according to whether the input is an image or a patch. Image-level segmentation-network-based methods could segment an image with only one forward pass. For example, Guo et al. proposed *BTS-DSN*, which uses multi-scale feature fusion and short connections to obtain fine vessel segmentation, and no postprocessing is required [21]. However, *BTS-DSN* use VGGNet as the backbone, and transfer learning is required. The main difficulty of accurate vessel segmentation lies in that it is hard to locate vessel boundaries accurately, and it is easy to identify tiny vessels as backgrounds. To deal with this problem, Wang et al. handled vessel segmentation from the perspective of hard-sample learning [23]. They designed an extra branch to learn hard labels individually, and the segmentation maps of multiple branches are fused to generate the final refined output. Jiang et al. incorporated scale attention mechanism into deep architectures, and proposed Bi-SANet for vessel segmentation [28]. Lin et al. proposed a high-resolution feature learning model to preserve spatial details [24]. However, all these models are huge, and there is still a lack of lightweight models.

Patch-level segmentation networks follow the pipeline of preprocessing, patch extraction, training/testing, and postprocessing [18,19,29]. For example, Yan et al. presented a three-stage segmentation network which extracts the thick and thin vessels in the first and the second stages, and finally fuses the former two stages’ features together in the third stage to generate the final segmentation map [29]. Wu et al. presented a patch-level two-stream network, called MS-NFN, for vessel segmentation [19]. In their method, the training sample consists of 190,000 overlapped patches of size 48 × 48. For testing, each test image was first preprocessed, and then overlapped patches with size 48 × 48 were extracted. At last, the segmentation probability maps of these image patches were recomposed to generate an entire segmentation map. There are many networks that belong to the patch-level segmentation networks, and the key characteristic of this kind of method is that they need multiple forward passes, rather than one forward pass, when testing.

Although state-of-the-art performance of these patch-level methods was achieved, patch extraction and reconstruction of segmentation maps are always required, resulting in low inference speed. Therefore, there is still lack of a fast image-level vessel segmentation network.

### 2.3. Lightweight Fundus Segmentation Models

Due to large number of parameters of VGGNet and ResNet, they are hard to deploy to edge side. To address this problem, some light-weighted network architectures have been proposed. For instance, MobileNet [30] and ShuffleNet [31] were proposed for image classification. MobileNet uses depthwise separable convolution rather than standard convolution to reduce parameters and accelerate inference. Meanwhile, it uses convolution with large stride to downsample; this setting makes the network achieve a large receptive field and could reduce computation. However, the setting of using convolution with a large stride is not suitable for vessel and lesion segmentation, since the spatial resolution of the feature maps are decreased during feature flow. For example, the resolution of the feature maps is only half of that of the input image after the first convolution operation in MobileNet. The resolution of feature maps was downsampled by a factor of 32 after Stage 4 in ShuffleNet. In our experiments, MobileNet was taken as a baseline.

As far as we know, there is still lack of a lightweight model for fundus segmentation. Guo et al. proposed a lightweight network, called LWENet, for fundus lesion segmentation [9]. However, there still exists the problem of losing spatial detailed information in the backbone of LWENet, and the LWENet relies on pretraining to achieve the claimed performance. Although there are some patch-level segmentation models with small model size, they show slow inference speed since they need multiple forward passes to generate a complete segmentation map for one test image, and this means that they are not suitable for mobile edge computing. In summary, there is still lack of a lightweight, accurate, and image-level fundus segmentation model for mobile edge computing.

## 3. LightEyes

**Figure 2:**
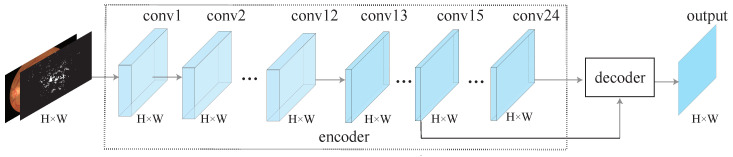
An overview of the proposed LightEyes. For simplicity, nonlinear activation functions (ReLU) are omitted. The encoder maintains a high resolution representation directly to facilitate the segmentation of tiny lesions and vessels. To be specific, the encoder contains 24 convolutional layers, and the decoder uses features of the intermediate layer to generate output.

The overview of the LightEyes is shown in Figure 2. As we can see, the LightEyes consists of two parts: the encoder and the decoder. The encoder mainly consists of 24 convolutional layers, from conv1 to conv24, and each convolution layer is followed by a ReLU activation function. The decoder makes use of multi-receptive field feature maps to generate the outputs. Detailed configurations of the LightEyes are summarized in Table 2.

### 3.1. Encoder

The encoder of the LightEyes has two characteristics: high-resolution feature representation, and small number of convolutional filters.

#### 3.1.1. Characteristic 1—High-Resolution Feature Representation

First of all, existing fundus lesion segmentation models achieve a large receptive field and extract semantic features by stacking multiple pooling operations, which lead to low-resolution feature representation. Continuous downsampling has no problem in image classification. However, in the field of tiny vessel segmentation in fundus images, there are some problems. The main problem is that with the forward propagation of features, the loss of spatial relationship between feature maps becomes more and more serious, specifically for small lesion points. As a result, the segmentation results obtained by upsampling are coarse and affect the diagnosis of the diseases. Most of the existing fundus segmentation models focused on designing complex feature fusion methods to alleviate this problem [8,13,21,32], such as the skip connection [14], atrous spatial pyramid pooling [33], and so on. Recent studies show that keeping high resolution of feature maps is helpful to boost segmentation performance [34,35], specifically for tiny objects, since high-resolution features convey more detailed spatial information than low-resolution features. For instance, low-resolution features are helpful to locate thick vessels, but it is hard to locate the vessel boundaries accurately. In contrast, high-resolution features could. More importantly, fundus structures usually have small sizes (the width of thin vessels may be as low as 1 pixel, and the areas of small microaneurysms are only a few pixels), thus it is necessary to learn high-resolution features. Therefore, a full-resolution encoder is designed in the LightEyes. In the encoder of the LightEyes, we stack 24 convolutional layers with kernel size 3 × 3 to achieve a large receptive field, and the field of view of the last convolutional layer can be 49 × 49. Therefore, the last several convolutional layers have larger receptive fields than those of the first several convolutional layers. As a result, the last several convolutional layers are expected to learn high-level discriminative features (semantic features) to facilitate the classification of non-vessels pixels and vessel pixels. In contrast, the first several convolutional layers have small receptive fields, and they convey less semantic information; thus, it is difficult to determine the category of pixels using only low-level features alone. In conclusion, the encoder of the LightEyes could not only extract semantic features but also maintain the high resolution of the feature map, so that the detailed information can be preserved in the encoder phase.

#### 3.1.2. Characteristic 2—Small Number of Convolutional Filters (at Most 16) for Each Convolutional Layer

Designing a high-resolution encoder for the segmentation of fundus images meets two problems: (1) High resolution of features corresponds to high memory usage. If VGGNet or ResNet does not use any downsampling operations, the high resolution of features cannot be maintained due to high memory cost. (2) For such an encoder with high resolution, there is no pretrained model available which could be used for fine-tuning. Therefore, the second problem is elucidating how to train such an encoder from scratch using dozens of fundus images.

In the LightEyes, each convolutional layer has at most 16 filters to solve above two problems. Considering the characteristics of the fundus segmentation, we think there is no need to have as many convolutional filters as in ImageNet-based natural image classification networks, such as VGGNet and ResNet. In those networks, a large number of convolution kernels are required to learn high-level object-related features. For example, to classify an image filled with a person, the network needs to consider various structures of the human body, such as head, arms, and legs. Therefore, numerous convolution kernels are required in the last few layers of the ImageNet-based classification networks. However, in the field of fundus structure segmentation, the prediction of a pixel does not need to consider twoabstract features, such as that in the classification networks. Segmentation of fundus structure mainly depends on color, texture, and context information. Therefore, in the encoder of LightEyes, the number of 3 × 3 kernels in one convolution layer is at most 16, and the total number of 3 × 3 kernels in the encoder is 288 (see Table 2).

Our experimental results show that the LightEyes could be trained effectively, even if no pretraining model is available, and the inference speed shows superior performance compared with other VGGNet-based or ResNet-based segmentation models.

### 3.2. Decoder

**Figure 3:**
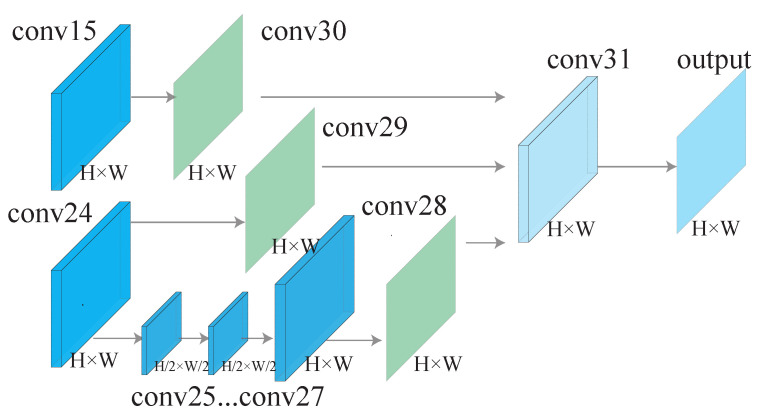
The decoder architecture. The input of the decoder is conv15 and conv24, which are learnt by the encoder. The decoder consists of three branches, and the output of three branches are further concatenated to generate the output of the LightEyes. Moreover, there are three supervision losses after conv28, conv29, and conv30.

The architecture of the decoder is visualized in Figure 3. We use multi-scale features in the decoder. The decoder consists of three branches. In the first branch, a convolutional layer (conv30) is performed after conv15 to generate a feature map with one channel. In the second branch, a convolutional layer (conv29) is performed after conv24 to generate a feature map with one channel. In the third branch, a pooling operation is first performed after conv24 to generate a low-resolution feature map, then the generated feature map is further processed by three convolutional layers, one upsampling operation, and one convolutional layer. Then, the outputs of the three branches are concatenated together to form a feature map of three channels. Further, followed by a convolution operation with kernel size 3 × 3 (conv31), a feature map of one channel is obtained. At last, after sigmoid function, a segmentation probability map is obtained. In the training phase, it is compared with ground truth to obtain a loss. In the testing phase, this probability map is actually the output of the LightEyes.

### 3.3. Implementation Details

#### 3.3.1. Deep Supervision

We add three auxiliary losses to the decoder of the LightEyes based on the following considerations. First, adding auxiliary losses to intermediate layers can obtain multiple segmentation results under different receptive fields [7]. Thereby, we can fuse these intermediate features together to obtain much better performance (see Figure 3). Second, we can explore the adequate receptive field in semantic segmentation of fundus images by comparing segmentation performance of the auxiliary loss. Third, different models can be deployed depending on the hardware sources of the mobile device. The three auxiliary losses correspond to three segmentation models, called LightEyes_A1 (the supervision loss is after conv30), LightEyes_A2 (the supervision loss is after conv29), and LightEyes_A3 (the supervision loss is after conv28). LightEyes_A1 (auxiliary loss1) contains the fewest parameters and the fastest inference speed, and it takes up the least amount of memory.

#### 3.3.2. Loss Function

Lesions and vessels account for a small proportion of the entire fundus image. For instance, the proportion of MA is about 0.1%. As a result, the number of background pixels is much larger than that of foreground pixels, which is called a class-imbalance problem. A solution to this problem is to introduce a weight factor β for foreground pixels and 1−β for background pixels; this method is called class-balanced cross-entropy loss [7]. Considering that this method does not consider easy/hard background pixels, which may incur misclassification, we use random drop loss [36] for training the LightEyes. The random drop loss is defined as

(1) $$L\left(p,y|\theta \right)=-\beta \sum_{y_{j}=1}\log p_{j}-\left(1-\beta \right)\sum_{y_{j}=0}𝟙\left(p_{j}\right)\log\left(1-p_{j}\right)$$

where *p* denotes a segmentation probability map obtained by sigmoid function, *y* denotes ground truth, and $\beta =\frac{N_{n}}{N_{n}+N_{p}}$ denotes the weight factor, where $N_{n}$ denotes the number of background pixels retained and $N_{p}$ denotes the number of foreground pixels. In addition, $𝟙(p_{j})$ is an indicator function to specify whether a background pixel is dropped or not according to its activation probability $p_{j}$, and it is defined below.

(2) $$𝟙\left(p_{j}\right)=\left\{\begin{array}{ccc} \; & 0,\; & r<p_{drop}\left(p_{j}\right)\\ \; & 1,\; & otherwise \end{array}\right.$$

where *r* is a random number and follows a uniform distribution between 0 and 1. In addition, $p_{drop}\left(\cdot \right)$ represents a random drop function that maps the activation probability to the drop probability. In our experiments, we use the linear drop function, namely, $p_{drop}\left(p_{j}\right)=1.0-p_{j}$. It is worthy noting that the three auxiliary losses and the fusion loss are the same as Equation (1).

## 4. Experiments

### 4.1. Materials

We evaluated the LightEyes over five publicly available datasets, i.e., IDRiD [37], e-ophtha EX [38], DRIVE [39], STARE [40], and CHASE_DB1 [41].

The IDRiD dataset consists of 81 images, each of which has a resolution of 2848×4288 (height × width), and pixel-wise annotations of MA and EX are provided. IDRiD provides a division of training set and testing set, of which 54 images are used for training and the remaining 27 for testing.

The e-ophtha EX dataset contains 82 fundus images, of which 47 images include EX. The resolutions of these images range from 960 × 1440 to 1696 × 2544 pixels. Considering that this dataset does not provide an explicit partition of training set and testing set, we adopt five-fold cross validation, as in [9], for a fair comparison with other models.

The DRIVE dataset is the most widely used dataset, and consists of 40 fundus images with resolution of 584 × 565. This dataset provides two groups of pixel-annotations for vessels. One is used as ground truth for training vessel segmentation algorithms, and the other one is used to compare the segmentation results between computer algorithms and the second human. This dataset provides a partition of training set and testing set, and each set contains 20 individual fundus images. The STARE dataset contains 20 color fundus images, each with resolution of 605 × 700. The CHASE_DB1 dataset consists of 28 fundus images with resolution of 960 × 999. However, the partition of training set and testing set is not provided for both STARE and CHASE_DB1. For fair comparison, we use the same partition as [19,21,29] on the CHASE_DB1, where the first 20 images are considered as training set and the remaining 8 images are considered as testing set. On the STARE dataset, after referring to [21,32,42], we used the first 10 images as training set, and the remaining 10 images as testing set.

### 4.2. Training Details

#### 4.2.1. Running Environments

The LightEyes was built based on an open-source framework Caffe [43]. Caffe was compiled with CUDA 8.0 and CUDNN 5.1, and it ran on a workstation with Ubuntu 18.04 operation system and NVIDIA GTX 1080ti GPUs.

#### 4.2.2. Training Data Preparation

The maximum resolutions of images in the IDRiD and e-ophtha EX datasets exceed 2000 pixels; therefore, we first scaled each image and its ground truth to 712×1072 (height × width) before training to reduce computational complexity, whereas on the DRIVE, STARE, and CHASE_DB1 datasets, no scaling was performed. In addition, we used various transformations to augment the training set, including rotation and flipping horizontally and vertically. As a result, there are 324, 120, 80, and 120 training images on the IDRiD, DRIVE, STARE, and CHASE_DB1 datasets, respectively.

#### 4.2.3. Parameter Settings

The parameters of the LightEyes were initialized using xavier [44]. The initial learning rate was set to $1\times 10^{-3}$, and we used bilinear interpolation for upsampling. For training the LightEyes, we used ADAM [45] to optimize the model. In our experiments, the LightEyes was trained using ADAM for 60 k/60 k/20 k/140 k/140 k iterations on the DRIVE/CHASE_DB1/STARE/IDRiD/e-ophtha datasets, respectively. A detailed comparison of LightEyes with other lesion segmentation models are summarized in Table 3.

### 4.3. Evaluation Metrics

We use sensitivity (Se), specificity (Sp), accuracy (Acc), Jaccard similarity index (JI), and the area under the ROC curve (AUC) to evaluate the vessel segmentation maps. In lesion segmentation task, we use precision (Pr), recall (Re), and F1-score (F1) to evaluate the lesion segmentation maps. They are defined as follows.

(3) Se=Re=TPTP+FN

(4) $$\mathrm{Sp}=\frac{\mathrm{TN}}{\mathrm{TN}+\mathrm{FP}}$$

(5) $$\mathrm{Pr}=\frac{\mathrm{TP}}{\mathrm{TP}+\mathrm{FP}}$$

(6) $$F1=\frac{2\times \mathrm{Pr}\times \mathrm{Se}}{\mathrm{Pr}+\mathrm{Se}}$$

(7) $$\mathrm{JI}=\frac{\mathrm{TP}}{\mathrm{TP}+\mathrm{FP}+\mathrm{FN}}$$

(8) $$\mathrm{Acc}=\frac{\mathrm{TP}+\mathrm{TN}}{\mathrm{TP}+\mathrm{FN}+\mathrm{TN}+\mathrm{FP}}$$

where true positive (TP) means foreground pixels (vessel, lesion) classified correctly, false negative (FN) denotes foreground pixels misclassified to background pixels, false positive (FP) denotes background pixels misclassified to foreground pixels, and true negative (TN) denotes background pixels classified correctly. TP, FN, FP, and TN are counted pixel-by-pixel, and when evaluating vessel segmentation results, we only count the pixels inside the field of view. To calculate these evaluation metrics, we select the equilibrium point (the closest point between Pr and Re) of the Pr–Re curve to binarize probability maps.

### 4.4. Comparison with State-of-the-Art Methods

To evaluate the effectiveness of the LightEyes, we employed experiments on the lesion segmentation and the vessel segmentation. Moreover, the LightEyes was verified on two kinds of lesions, including the microaneurysm and the hard exudate.

#### 4.4.1. Lesion Segmentation

We compare our LightEyes with several state-of-the-art lesion segmentation methods: HED [7], FCRN [13], L-Seg [8], and LWENet [9]. The performance of these methods and the LightEyes on lesion segmentation are summarized in Table 4, where the performance of HED and FCRN are reported in [8,9]. We can observe that the LightEyes outperforms other models in terms of all metrics. Moreover, on EX segmentation, the LightEyes shows superior performance to three models, namely, FCRN, L-Seg, and LWENet, which are specifically designed for fundus lesion segmentation.

**Figure 4:**
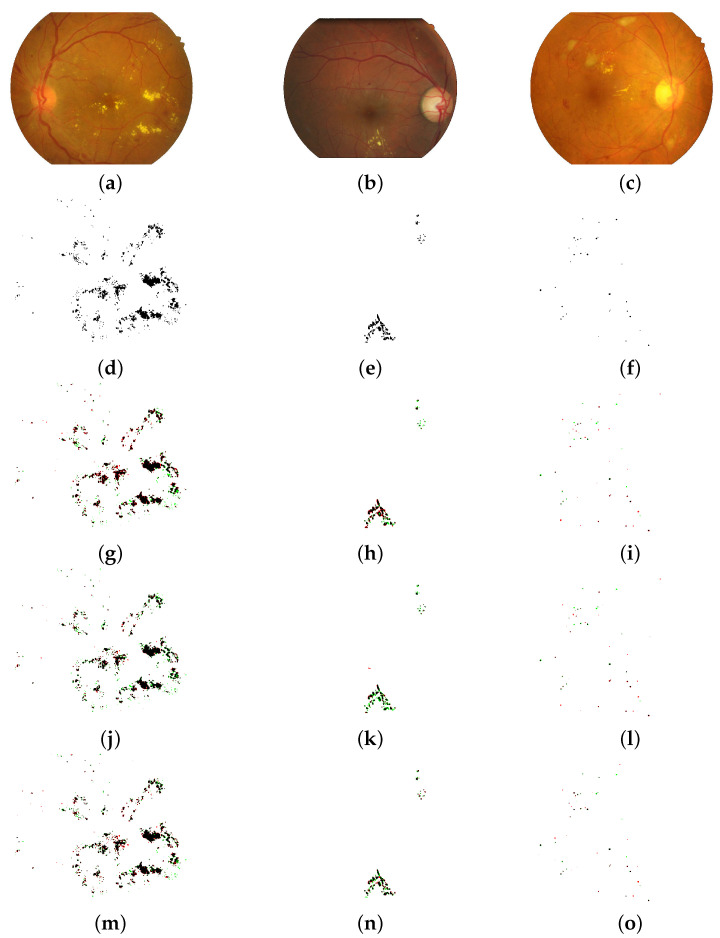
Lesion segmentation binary maps of the LightEyes and other methods (TPs are marked as black, FPs are marked as red, and FNs are marked as green). The first and the second column denote EX segmentation on the IDRiD dataset, and the third row denotes MA segmentation on the IDRiD datasets. (**a**) fundus image (IDRiD). (**b**) fundus image (e-ophtha EX). (**c**) fundus image (IDRiD). (**d**) ground truth (EX). (**e**) ground truth (E1). (**f**) ground truth (M1). (**g**) HED (F1 = 0.8153, JI = 0.6881). (**h**) HED (F1 = 0.7645, JI = 0.6192). (**i**) HED (F1 = 0.4950, JI = 0.3280). (**j**) LWENet (F1 = 0.8396, JI = 0.7236). (**k**) LWENet (F1 = 0.7104, JI = 0.5529). (**l**) L-Seg (F1 = 0.5367, JI = 0.3668). (**m**) LightEyes (F1 = 0.8433, JI = 0.7289). (**n**) [LightEyes (F1 = 0.7827, JI = 0.6457). (**o**) LightEyes (F1 = 0.6177, JI = 0.4468).

We conclude that the LightEyes has the following advantages on fundus lesion segmentation over state-of-the-art approaches. (1) The LightEyes achieves the best segmentation performance on all datasets. In the LightEyes, less detailed information is lost due to a large receptive field obtained by stacking multiple convolutional layers and one pooling operation. As a result, the LightEyes achieves much higher Se than HED and L-Seg. From Figure 4, we can observe that the segmentation maps of the LightEyes are much finer compared with HED. Compared with LWENet, they contain too many FNs and the boundaries are not clear. (2) The inference speed of the LightEyes is the fastest due to its low computation cost and simple architecture. (3) Our proposed LightEyes achieves superior performance with the least number of parameters. Specifically, the number of parameters of LightEyes is only 0.25% of VGGNet-based models, namely, HED and L-Seg. Compared with a lightweight network, LWENet, our method also behaves better in model size. (4) LightEyes is the only model trained from scratch. No pretraining is required, which means that the LightEyes can achieve good segmentation performance using only dozens of annotated fundus images.

**Figure 5:**
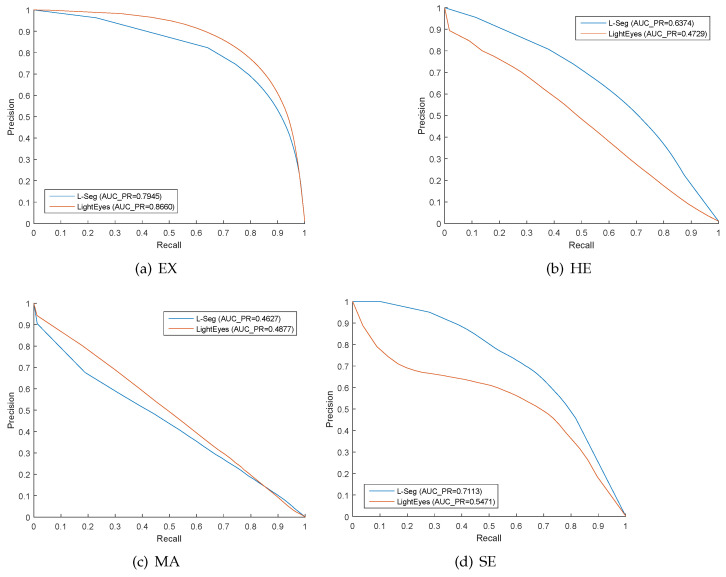
P–R curves for hard exudate (EX), hemorrhage (HE), microaneurysms (MA), and soft exudate (SE) on IDRiD dataset.

At last, the precision–recall curves of LightEyes in the segmentation of hard exudate, microaneurysms, hemorrhage, and microaneurysms over the IDRiD dataset are visualized in Figure 5.

#### 4.4.2. Vessel Segmentation

We compare the LightEyes with other state-of-the-art vessel segmentation methods on the DRIVE, CHASE_DB1, and STARE datasets, and the results are shown in Table 5, Table 6 and Table 7. Meanwhile, Table 8 shows detailed comparisons of the LightEyes and other methods.

As we can see from Table 5, the LightEyes achieves the best vessel segmentation performance among all image-level models, namely, DRIU and BTS-DSN. Compared with patch-level models, the LightEyes shows competitive performance with three-stage FCN, MS-NFN, and DUNet. Although the segmentation performance of the LightEyes is worse than that of FCN [18], the fps of the LightEyes is 100× that of FCN. As shown in Table 6, on the CHASE_DB1 dataset, the LightEyes achieves the highest accuracy compared with other methods, whether patch-level or image-level. Moreover, the fps of the LightEyes is over 900× that of DUNet.

We can observe from Table 8 that the LightEyes is the only image-level segmentation model that does not require pretraining, preprocessing, and postprocessing. For patch-level models, they need preprocessing to split the raw image into a few patches, and postprocessing is needed to reconstruct vessel maps. As a result, they segment a fundus image in the slow speed. For the LightEyes, these additional processes are not required. Meanwhile, the LightEyes shows big advantages in speed. Compared with MS-NFN and DUNet, the LightEyes achieves comparable performance with over 500 × speed.

Meantime, we can observe from Table 8 that LightEyes achieves comparable performance with the minimum number of parameters. Specifically, the number of parameters of LightEyes is only 0.46% of VGGNet-based models, namely, DRIU and BTS-DSN.

**Figure 6:**
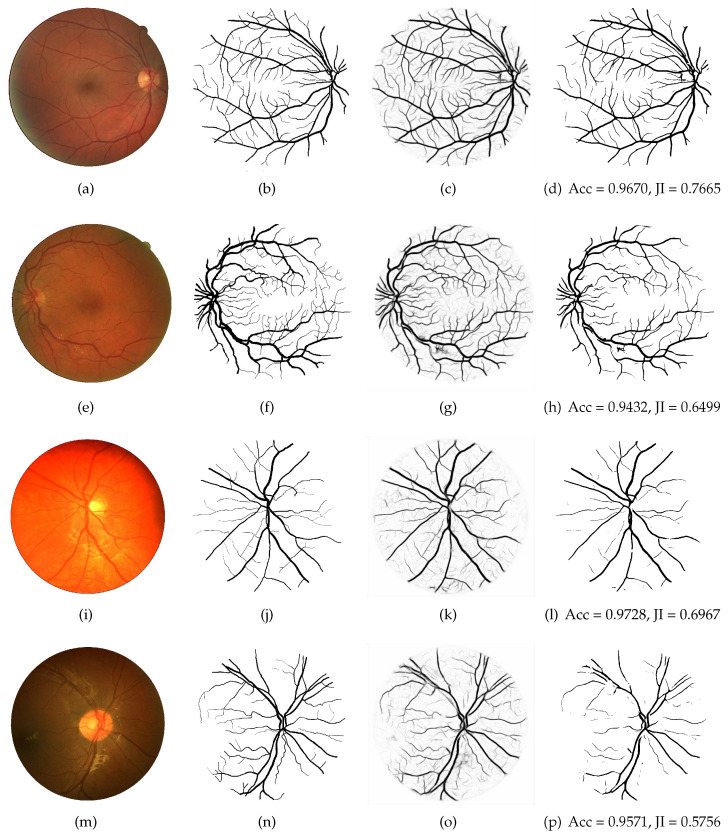
Examples of the best and worst vessel segmentation results by the proposed the LightEyes on images from the DRIVE and CHASE_DB1 datasets. The first and second rows correspond to the highest and lowest accuracy on the DRIVE, and the third and fourth rows correspond to the highest and lowest accuracy on the CHASE_DB1. From column 1 to 4: original fundus images (**a**,**e**,**i**,**m**), ground truth (**b**,**f**,**j**,**n**), probability maps (**c**,**g**,**k**,**o**) and binary maps (**d**,**h**,**l**,**p**).

At last, segmentation maps of the LightEyes are visualized in Figure 6. As can be seen, the proposed method could detect both thin and thick vessels.

### 4.5. Comparison with MobileNet

We compare the segmentation performance of the LightEyes with MobileNet [30], which is designed specificity for mobile devices.

In our experiments, we designed MobileNet-Fundus for fundus segmentation. The encoder of the MobileNet-Fundus is the first of four stage operations of the MobileNet, and its decoder is similar to LightEyes, i.e., three deep supervision losses at different stages and weighted feature fusion. For fair comparison, we use the same loss function, training samples, and optimization method. The results are summarized in Table 9 and Table 10.

We can observe from these tables that LightEyes achieves much better segmentation performance on all experiments; in particular, the sensitivity and F1-score of the LightEyes are much higher than these of the MobileNet-Fundus, and this means that the LightEyes can detect more thin vessels and small lesions. Moreover, the inference speed of the LightEyes is faster than the MobileNet-Fundus on the DRIVE and STARE datasets. Although the inference speed of the MobileNet-Fundus is faster than LightEyes on the IDRiD dataset, the architecture of the MobileNet-Fundus is unfriendly to small microaneurysms segmentation since it is essential to keep high resolution of the feature maps in the encoder.

Moreover, we can observe from Table 8 that the number of parameters of LightEyes is one-eighth of that of MobileNet-Fundus, while the segmentation accuracy is over 1.8% higher than MobileNet-Fundus. In summary, the proposed LightEyes shows better segmentation performance and inference speed than MobileNet-Fundus and LWENet.

### 4.6. Performance on the Edge Devices

We took experiments on three edge devices: Cambricon-1A, Cambricon-MLU100, and Cambricon-MLU270.

Cambricon-1A chip is a mobile edge device specially for deep learning, which was launched by Cambrian in 2017. It has the characteristics of high performance, low power consumption, and small area. The chip can be widely used in all kinds of intelligent terminals, including smart phones, security monitoring, and so on. Cambricon-1A has the highest working frequency of 1 GHZ, the maximum power consumption of about 18 W, the peak operation speed of 3 TOPS, and the maximum support of 2 GB DDR3 memory. Cambricon-MLU270 is a cloud acceleration chip for artificial intelligence applications. Its maximum power consumption is about 70 W, and the TOPS for int8 is about 128. In addition, it is equipped with 16 GB DDR4 memory.

The performances of the LightEyes and other models on Cambricon-1A are summarized in Table 11. We can observe that VGGNet-based segmentation models cannot handle large resolution images (1280 × 1280) due to large memory consumption, but the LightEyes could process these images. Moreover, the speed of the LightEyes in processing lower resolution images (960 × 999) is two to three times that of VGGNet-based models. For ResNet-based networks, such as FCRN, the speed is about the same as the LightEyes. However, the segmentation performance of FCRN is much lower than the LightEyes or even LightEyes_A1, whose speed is three times that of FCRN. Moreover, we can observe that segmenting a fundus image with size 712 × 1072 or 960 × 999 takes up to 2 s, and this could meet clinical needs of real-time processing.

When segmentation models are deployed to Cambricon-MLU270, we can observe that VGG-based models show the fastest inference speed and ResNet-based models show the slowest inference speed, and the proposed LightEyes only behaves slightly better than FCRN. We think the reason is that the dense convolution operations have been well accelerated on the MLU270, while for ResNet with skip connections, the acceleration effect is not as obvious as the VGGNet.

## 5. Discussion

### 5.1. Ablation Study on the Number of Filters

We trained the LightEyes with different number of convolutional filters to show that more filters have little effect on the improvement of segmentation performance, and it may cause training failure. We can observe from Table 12 that when the number of filters increased from 16 to 64, the model failed to converge on all experiments. We think that this may be due to the number of parameters increasing sharply (over 20×), while the number of training images remains unchanged. Moreover, the AUC increases slightly or even decreases (from 0.9829 to 0.9805) on the CHASE_DB1 dataset. Moreover, compared with LightEyes, the number of parameters and the inference speed could be further improved when NC was set to 8. However, if NC was set to 4, the segmentation performance decreased dramatically, but the network can still learn discriminative features. This part of the experiment validates our design principle to choose a small number of convolutional filters, and the optimal number may depend on the specific task.

### 5.2. Ablation Study on the Network Depth

We trained the LightEyes with different network depth to explore the suitable network depth on the segmentation of fundus structures. We stacked a series of convolutional operations with 3 × 3 kernel directly, and each convolutional layer contains 16 filters and is followed by a ReLU operation. At the last convolutional layer, a convolutional layer with 1 × 1 kernel was performed to generate segmentation maps, which was further fed into the loss function. The experimental results are summarized in Table 13. We can observe that the optimal network depth depends on the segmentation task, such as, for the vessel segmentation task, on average, 24 layers are more appropriate. However, for the lesion segmentation task, a deeper network will fail training due to the increase in the amount of parameters. Meanwhile, we conclude that it is possible to train a deep network with no downsampling for the segmentation of fundus structures, and this kind of network could also achieve comparable performance.

### 5.3. Performance of Auxiliary Losses

The segmentation performance of multiple receptive fields is shown in Table 14. We can observe from Table 14 that when the receptive field increases from 33 × 33 (auxiliary loss1) to 51 × 51 (auxiliary loss2), the performance is improved significantly in terms of lesion segmentation and vessel segmentation. However, when the performance of auxiliary loss2 is compared with auxiliary loss3, the increase in segmentation performance becomes smaller. We think a 51 × 51 receptive field is adequate for lesion segmentation and vessel segmentation. In addition, we can observe that the LightEyes achieves the highest metrics compared with LightEyes_A1, LightEyes_A2, and LightEyes_A3, which demonstrates the effectiveness of feature fusion. Meanwhile, we find that the performance of LightEyes_A1 is not bad, especially on lesion segmentation, which means it can also be deployed to mobile sides with faster speed.

### 5.4. Less Training Samples

In this section, we will explore how the LightEyes behaves when only part of the images sampled from the training set are involved in training. We employed two groups of experiments; one is the EX segmentation on the IDRiD dataset, and the other one is the vessel segmentation on the DRIVE dataset. On the IDRiD dataset, we sampled 9, 18, 27, 36, and 45 images for experiments, and we sampled 5, 10, and 15 images on the DRIVE dataset. The experimental results are summarized in Table 15 and Table 16. We can observe that when only 27 (50%) images participate in training, the LightEyes obtains an F1-score of 0.7847, which is very close to 0.7937 (100%). For vessel segmentation, the LightEyes also behaves well when using only five images, as can be observed in Table 16. This part of the experiment shows that even with only a small amount of annotated images, our model can learn well and obtains good generalization performance.

### 5.5. Performance on Optic Disc Segmentation

Previous experiments over fundus vessels and lesions reveal that the proposed method shows competitive performance compared with several deep segmentation models. In this section, experiments were conducted on the segmentation of optic discs (OD), which usually have much larger sizes than fundus vessels and lesions. The Messidor [47] dataset was used in the experiments. Almazroa et al. [48] annotated the optic disc boundary of 460 fundus images in the Messidor dataset. We used 400 images for model training and the remaining 60 images for testing. In addition, Dice index was used for performance evaluation. The segmentation performance of the LightEyes and other models is summarized in Table 17.

As can be seen, the proposed LightEyes has a higher Dice index than DeepLab-v2, but it is lower than UNet. The reason behind this may be that LightEyes has insufficient ability in learning high-level features, since the motivation of LightEyes is preserving spatially detailed information to boost the segmentation of tiny objects.

## 6. Conclusions

In this paper, we present a lightweight model, called the LightEyes, for the segmentation of fundus images on the mobile side. Considering the small size of fundus lesions and other structures, we design a high-resolution encoder, and each convolutional layer has less than 16 filters to decrease training difficulty since no pretraining model is available. Compared with other methods in lesion segmentation and vessel segmentation, the LightEyes shows comparable, or even superior, performance in terms of segmentation accuracy, inference speed, and memory usage. Specially, in fundus vessel segmentation, the speed of the LightEyes is up to 3–700× faster than other models. Meantime, the LightEyes achieves better segmentation accuracy than other deep models. Compared with MobileNet, the proposed LightEyes achieves higher segmentation accuracy with very close, or even faster, inference speed, achieving better accuracy–speed trade-off. Moreover, we deployed the LightEyes to mobile devices. Results show that the LightEyes could not only process large-resolution images, but also show high segmentation accuracy. The source code will be released at https://github.com/guomugong/LightEyes (accessed on 1 March 2022).

## Figures and Tables

**Table 2:** Configurations of the LightEyes (H and W below represent the height and width of the input image).

	Layer	Input Size	Output Size	#Filters	#Param	Ops (H × W)
Encoder	conv1	H × W × 3	H × W × 16	16	448	512
	conv*i* (i∈[2,12])	H × W × 16	H × W × 16	16	2320	2800
	conv13	H × W × 16	H × W × 8	8	1160	1400
	conv*i* (i∈[14,24])	H × W × 8	H × W × 8	8	584	696
Decoder	Pooling	H × W × 8	H/2 × W/2 × 8	-	0	-
	conv*i* (i∈[25,27])	H/2 × W/2 × 8	H/2 × W/2 × 8	8	584	174
	conv28	H/2 × W/2 × 8	H/2 × W/2	1	73	21.75
	conv29	H × W × 8	H × W	1	73	87
	conv30	H × W × 8	H × W	1	73	87
	conv31	H × W × 3	H × W	1	28	32
	total			316	35,551	41,117.75

**Table 3:** Hyperparameter settings of LightEyes and comparison methods.

Methods	Dataset	Optimizer	Learning Rate	Iteration	Batch Size
L-Seg [8]	IDRiD	SGD	$1\times 10^{-9}$	25,000	1
	e-ophtha EX	SGD	$1\times 10^{-8}$	20,000	1
HED [7]	IDRiD	SGD	$1\times 10^{-8}$	25,000	1
	e-ophtha EX	SGD	$1\times 10^{-8}$	20,000	1
LWENet [9]	IDRiD	SGD	$1\times 10^{-8}$	160,000	1
	e-ophtha EX	SGD	$1\times 10^{-8}$	160,000	1
LightEyes	IDRiD	ADAM	$1\times 10^{-3}$	140,000	1
	e-ophtha EX	ADAM	$1\times 10^{-3}$	140,000	1
	DRIVE	ADAM	$1\times 10^{-3}$	60,000	1
	CHASE_DB1	ADAM	$1\times 10^{-3}$	60,000	1
	STARE	ADAM	$1\times 10^{-3}$	20,000	1

**Table 4:** Comparison of the LightEyes and other models on lesion segmentation (fps = $1/t_{i}$, where $t_{i}$ is the inference time (exudate IO) of the model when the input image size of the model is 960 × 1440).

Model	Pre-Train	#Params(M)	fps	MA (IDRiD)			EX (IDRiD)			EX (e-ophtha EX)		
				Pr	Re	F1	Pr	Re	F1	Pr	Re	F1
HED [9]	Y	14.3	5.9	0.4291	0.4799	0.4474	0.7414	0.7618	0.7515	0.5049	**0.5727**	0.5336
FCRN [9]	Y	22.5	7.1	0.3542	0.3312	0.3423	0.6018	0.6862	0.6412	0.3807	0.5073	0.4326
L-Seg [8]	Y	14.3	5.9	0.4677	0.4720	0.4698	0.7436	0.7479	0.7457	–	–	–
LWENet [9]	Y	1.9	11.1	0.4221	0.4162	0.4191	0.7826	0.7803	0.7815	0.4812	0.5147	0.4960
LightEyes	N	**0.036**	**14.3**	**0.4960**	**0.4936**	**0.4948**	**0.7940**	**0.7933**	**0.7937**	**0.5409**	0.5424	**0.5417**

–: Not Reported.

**Table 5:** Segmentation results of vessel on the DRIVE dataset (best results shown in bold).

Input	Model	Se	Sp	Acc	AUC	JI	fps
Image	DRIU [32]	0.7855	0.9799	0.9552	0.9793	0.6904	15.1
	BTS-DSN [21]	0.7800	**0.9806**	0.9551	**0.9796**	0.6884	14.3
	LightEyes	**0.7896**	0.9805	**0.9562**	**0.9796**	**0.6965**	**51.3**
Patch	Three-stage FCN [29]	0.7631	0.9820	0.9538	0.9750	0.6793	–
	MS-NFN [19]	0.7844	0.9819	0.9567	0.9807	0.6978	0.1
	FCN [18]	0.8039	0.9804	0.9576	0.9821	0.7087	0.5
	DUNet [22]	0.7963	0.9800	0.9566	0.9802	0.7003	0.07
	Vessel-Net [46]	0.8038	0.9802	0.9578	0.9821	0.7077	–

–: Not Reported.

**Table 6:** Segmentation results of vessel on the CHASE_DB1 dataset (best results shown in bold).

Input	Model	Se	Sp	Acc	AUC	JI	fps
Image	BTS-DSN [21]	**0.7888**	0.9801	0.9627	**0.9840**	0.6579	11.7
	LightEyes	0.7709	**0.9841**	**0.9647**	0.9829	**0.6651**	**19.6**
Patch	Three-stage FCN [29]	0.7641	0.9806	0.9607	0.9776	0.6399	–
	MS-NFN [19]	0.7538	0.9847	0.9637	0.9825	0.6538	–
	DUNet [22]	0.8155	0.9752	0.9610	0.9804	0.6534	0.02
	Vessel-Net [46]	0.8132	0.9814	0.9661	0.9860	0.6857	–

–: Not Available.

**Table 7:** Segmentation results of vessel on the STARE dataset (best results shown in bold).

Input	Model	Se	Sp	Acc	AUC	JI	fps
Image	DeepVessel [42]	0.7412	–	0.9585	–	–	–
	BTS-DSN [21]	**0.8201**	0.9828	**0.9660**	**0.9872**	**0.7147**	10.6
	DRIU [32]	0.8036	0.9845	0.9658	0.9773	0.7097	11.6
	LightEyes	0.7830	**0.9864**	0.9653	0.9820	0.7012	**43.5**

–: Not Available.

**Table 8:** Comparison of proposed method and other vessel segmentation models (in the table, fps = $1/t_{i}$, where $t_{i}$ is the forward propagation time of a single image with size 584 × 565).

Model	Input	Pre-Train	Pre-Process	Post-Process	#Params (M)	fps	Acc		
							DRIVE	CHASE_DB1	STARE
Three-stage FCN [29]	patch	No	Yes	Yes	20.4	–	0.9538	0.9607	–
Vessel-Net [46]	patch	No	Yes	Yes	–	–	**0.9578**	**0.9661**	–
MS-NFN [19]	patch	No	Yes	Yes	0.4	0.1	0.9567	0.9637	–
DUNet [22]	patch	No	Yes	Yes	0.9	0.07	0.9566	0.9610	–
FCN [18]	patch	No	Yes	Yes	0.2	0.5	0.9576	–	–
DRIU [32]	image	Yes	No	No	7.8	15.1	0.9552	–	0.9658
BTS-DSN [21]	image	Yes	No	No	7.8	14.3	0.9551	0.9627	**0.9660**
MobileNet-Fundus	image	Yes	No	No	0.27	43.5	0.9518	0.9560	0.9473
LightEyes	image	No	No	No	**0.036**	**51.3**	0.9562	0.9647	0.9653

–: Not Reported.

**Table 9:** Comparison with MobileNet on vessel segmentation (Best results are bold).

Dataset	Model	Se	Sp	Acc	AUC	JI	fps
DRIVE	MobileNet-Fundus	0.6922	0.9772	0.9518	0.9570	0.5986	43.5
	LightEyes	**0.7896**	**0.9805**	**0.9562**	**0.9796**	**0.6965**	**51.3**
CHASE_DB1	MobileNet-Fundus	0.7006	0.9817	0.9560	0.9722	0.5922	**25.0**
	LightEyes	**0.7709**	**0.9841**	**0.9647**	**0.9829**	**0.6651**	19.6
STARE	MobileNet-Fundus	0.6522	0.9816	0.9473	0.9604	0.5633	37.6
	LightEyes	**0.7830**	**0.9864**	**0.9653**	**0.9820**	**0.7012**	**43.5**

**Table 10:** Comparison with MobileNet on lesion segmentation (Best results are bold).

Lesion	Model	Pr	Re	F1	JI	fps
MA	MobileNet-Fundus	0.4072	0.4040	0.4056	0.2544	**19.1**
	LightEyes	**0.4960**	**0.4936**	**0.4948**	**0.3287**	14.3
EX(IDRiD)	MobileNet-Fundus	0.7418	0.7381	0.7400	0.5872	**19.1**
	LightEyes	**0.7940**	**0.7933**	**0.7937**	**0.6579**	14.3

**Table 11:** Fps of the LightEyes and other models on Cambricon-1A and Cambricon-MLU270.

Hardware	Resolution	VGGNet-Based		ResNet-Based	LightEyes	LightEyes_A3	LightEyes_A2	LightEyes_A1
		L-Seg	BTS-DSN	FCRN				
Cambricon-1A	584 × 565	0.5	0.5	1.7	1.6	2.6	3.2	4.2
	712 × 1072	0.2	0.2	0.7	0.7	1.2	1.4	1.9
	960 × 999	0.2	–	0.5	0.6	0.9	1.1	1.6
	1280 × 1280	–	–	0.3	0.3	0.6	0.6	0.9
Cambricon-MLU270	584 × 565	7.9	7.3	3.4	3.9	4.5	6.4	9.5
	712 × 1072	3.1	2.8	1.5	1.7	1.9	2.8	4.1
	960 × 999	2.6	2.2	1.2	1.3	1.5	2.2	3.2
	1280 × 1280	1.5	1.3	0.7	0.8	0.9	1.3	1.9
Cambricon-MLU100	584 × 565	1.9	1.7	4.8	4.8	6.0	6.3	8.5
	712 × 1072	0.8	0.6	2.4	2.1	2.6	2.8	3.8
	960 × 999	0.7	0.5	1.9	1.7	2.1	2.2	3.0
	1280 × 1280	0.4	0.3	1.1	1.0	1.1	1.2	1.7

–: out of memory.

**Table 12:** Segmentation results when varying the number of filters in LightEyes (NC denotes the number of convolutional filters per convolution layer in the backbone of the LightEyes, and we use AUC/F1-score to evaluate vessel/lesion segmentation maps). Best results are bold.

Datasets	NC = 2	NC = 4	NC = 8	LightEyes	NC = 32	NC = 48	NC = 64
DRIVE	–	0.9734	0.9794	0.9796	0.9796	**0.9797**	–
CHASE_DB1	0.9535	0.9722	0.9798	**0.9829**	0.9818	0.9805	–
STARE	0.9359	0.9744	0.9798	0.9820	**0.9863**	0.9843	–
IDRiD (EX)	0.6741	0.7775	0.7868	**0.7937**	–	–	–
IDRiD (MA)	–	0.4769	**0.4956**	0.4948	–	–	–

–: failed to train.

**Table 13:** Segmentation results when varying the network depth (NL denotes the number of convolutional layers in the backbone of the LightEyes, and we use AUC/F1-score to evaluate vessel/lesion segmentation maps). Best results are bold.

Datasets	NL = 6	NL = 12	NL = 18	NL = 24	NL = 30	NL = 36
DRIVE	0.9703	0.9746	0.9754	**0.9770**	0.9752	0.9735
CHASE_DB1	0.9539	0.9739	0.9751	0.9763	0.9737	**0.9769**
STARE	0.9419	0.9650	0.9634	0.9729	**0.9781**	0.9681
IDRiD (EX)	0.7452	0.7610	**0.7849**	0.7689	–	–
IDRiD (MA)	0.4618	**0.4711**	0.4669	–	–	–

–: failed to train.

**Table 14:** Comparison of segmentation results and the number of parameters (Fps was measured under input with size 712 × 1072 and 584 × 565 on the IDRiD and DRIVE). Best results are bold.

Model	Params	MA				EX(IDRiD)			DRIVE				
		Pr	Re	F1	fps	Pr	Re	F1	Se	Sp	Acc	AUC	fps
LightEyes_A1	28369	0.4864	0.4797	0.4830	**36.4**	0.7533	0.7561	0.7547	0.7769	0.9800	0.9540	0.9760	**79.4**
LightEyes_A2	33625	0.4913	**0.4973**	0.4943	27.8	0.7833	0.7855	0.7844	0.7849	0.9808	0.9558	0.9788	58.5
LightEyes_A3	35377	0.4903	0.4935	0.4919	27.0	0.7937	0.7932	0.7934	0.7783	**0.9814**	0.9555	0.9788	56.2
LightEyes	35551	**0.4960**	0.4936	**0.4948**	25.6	**0.7940**	**0.7933**	**0.7937**	**0.7896**	0.9805	**0.9562**	**0.9796**	51.3

**Table 15:** The performance of the LightEyes when varying the number of training samples on the hard exudate segmentation task (IDRiD dataset).

Proportion	Pr	Re	F1
16.7% (9/54)	0.7483	0.7488	0.7486
33.3% (18/54)	0.7771	0.7762	0.7766
50% (27/54)	0.7842	0.7852	0.7847
66.7% (36/54)	0.7841	0.7838	0.7839
83.3% (45/54)	0.7843	0.7820	0.7831
100% (54/54)	0.7940	0.7933	0.7937

**Table 16:** The performance of the LightEyes when varying the number of training samples on the vessel segmentation task (DRIVE dataset).

Proportion	Se	Sp	AUC
25% (5/20)	0.7713	0.9799	0.9757
50% (10/20)	0.7758	0.9810	0.9779
75% (15/20)	0.7796	0.9806	0.9784
100% (20/20)	0.7896	0.9805	0.9796

**Table 17:** The performance of the LightEyes and comparison with other methods on the Messidor dataset.

Models	DeepLab-v2 [33]	UNet [49]	LightEyes
Dice Index	0.9197	0.9567	0.9358

## Data Availability

The datasets used in this work are publicly available online.
